# Fellow-Workers and Their Doings

**Published:** 1914-08-15

**Authors:** 


### ROUND THE WORLD.

### Fellow-Workers and their Doings.

[The Editor Invites Early Information and Contributions Marked "Fellow-Workers."]

The new medical superintendent of the State Institution for Mental Defectives at Moss Side, Liverpool, Dr. William Rees Thomas, studied medicine at Cardiff and Charing Cross Hospital, subsequently becoming an M.D. Lond., M.R.C.S., L.R.C.P. Having added to his academic successes by taking the Murchison Scholarship of the Royal College of Physicians two years ago, Dr. Rees Thomas turned his attention to psychological medicine, and won the Gaskell Prize and Gold Medal, as well as the Certificate of the Medico-Psychological Association last year. He subsequently obtained the appointment of senior assistant medical officer and pathologist to the East Sussex County Asylum, Hellingly.

The medical profession is often indebted for new ideas to scientific co-workers outside its ranks, and Mr. J. Arthur Thompson, M.A., LL.D., who recently delivered the popular lecture at the British Medical Association's Annual Congress, has certainly put the *vis medicatrix naturæ* in a new light. Indeed, few practitioners will have heard of or read the report of this address without realising a deeper sense of reverence for the mystery that lies behind all natural phenomena, particularly in regard to the capacity living organisms have for self-healing. Mr. Thompson is, of course, the well-known Regius Professor of Natural History at Aberdeen University.

Mr. Percival P. Cole, who has just been appointed assistant surgeon to the Cancer Hospital, originally entered Guy's as a dental student, taking the L.D.S. of the Royal College of Surgeons in 1899. Subsequently taking up medicine, he qualified M.R.C.S., L.R.C.P. after five years' work, and became an F.R.C.S. Eng. in 1906, further adding to his academic distinctions by taking the M.B., Ch.B. of Birmingham University a few years later. Mr. Cole has been successful in obtaining surgical appointments in London, and at the present time is assistant surgeon to the *Dreadnought* Hospital, Greenwich, and surgeon to the West Ham and East London Hospital. His appointment at the Cancer Hospital is a recognition of the good work he has put in there as surgical registrar. Amongst past appointments he has held may be mentioned those of demonstrator in anatomy at the Middlesex Hospital and a similar post at Birmingham.

Another "Bart.'s" man, Dr. Ray Edridge, has been elected to the post of assistant anæsthetist at the Royal United Hospital, Bath. Mr. Edridge qualified M.R.C.S., L.R.C.P. in 1905, and after holding the appointments of ophthalmic surgeon at Guy's, house physician to the Evelina Hospital, and clinical assistant at the East London Children's Hospital he settled in Bath and became medical officer to the Southern Dispensary there. He was at one time editor of the *Guy's Hospital Gazette.*

Mr. D. P. Dalbreck Wilkie, who at the recent meeting of the British Medical Association made an interesting suggestion that persons affected by status lymphaticus bear appendicitis and other acute abdominal conditions exceptionally badly, is an energetic member of the younger generation of Edinburgh surgeons. He has worked hard at the University, the Royal Infirmary, and other institutions there. Becoming an F.R.C.S. and M.D. of Edinburgh some six years ago, he was subsequently awarded the gold medal at the Ch.M. examination. Mr. Wilkie's present appointments include those of assistant surgeon to the Edinburgh Royal Infirmary and assistant surgeon to the Leith Hospital. He has written a good deal on the pathology and treatment of various abdominal conditions.

Mr. Christopher Kempster, L.R.C.P., M.R.C.S., who has given special attention to the treatment of rodent ulcer by *x*-rays, summarises his experiences and conclusions in an interesting communication to the *Practitioner* (August 1914). As he truly points out, although everyone is familiar with rodent ulcer, the pathology of this condition is quite unknown, and consequently in this instance the surgeon obtains no help from the laboratory. However, as to treatment, Mr. Kempster has formed the opinion that the *x*-ray method is unquestionably superior to all other measures, not excluding excision, the cautery, scraping, cataphoresis, and electrolysis. He particularly points out that after *x*-ray treatment as good a result is obtained from the cosmetic point of view as could be possibly expected. Mr. Kempster is physician to the *x*-ray and electro-therapeutic department at St. John's Hospital for Skin Diseases in Leicester Square, where he also lectures on his special subject, and assistant in the *x*-ray department at the Cancer Hospital.

Further investigations to be subsidised by the Government out of its special annual medical grant will include research in connection with the tuberculin skin reactions obtained in cases of bone and joint tuberculosis to be carried out by Mr. H. J. Gauvain, the medical superintendent of the Lord Mayor Treloar Cripples' Hospital at Alton. Mr. Gauvain won scholarships both at Cambridge and St. Bartholomew's, and after qualifying B.C. Cantab., M.R.C.S., L.R.C.P., and holding resident appointments worked for some time as clinical assistant in the orthopædic department at "Bart's."

Under the same ægis Major Harrison, R.A.M.C., is to pursue a detailed examination of the Wassermann reaction, but it may be supposed that the Sub-Committee of the Royal Society of Physicians, with whom he is to collaborate, will not see much of his services until the end of the war. Major Harrison, who is a medical graduate of Glasgow University, holds the important post of assistant professor of pathology at the Royal Army Medical College.

At the same time provision is to be made for Drs. Mellanby and Twort to continue the researches on infantile diarrhœa, upon which they have been engaged for some time. The investigations of these two representatives of scientific medicine are specially directed towards a better understanding of intestinal toxic absorption.

The Government grant in question will also be used to assist Drs. Dale and C. J. Lewis in furthering their investigations as to the cause of still-birth, a problem of considerable medical importance.

